# Modifying Sensory Afferences on Tablet Changes Originality in Drawings

**DOI:** 10.3389/fpsyg.2022.806093

**Published:** 2022-06-14

**Authors:** Fabien Bitu, Béatrice Galinon-Mélénec, Michèle Molina

**Affiliations:** ^1^Laboratory of Psychology of Caen Normandy (LPCN EA7452), University of Caen Normandy, Caen, France; ^2^Identity and Differentiation of Spaces, Environment and Societies (UMR IDEES 6266 CNRS), University Le Havre Normandy, Le Havre, France

**Keywords:** creativity, tablet, children, adolescent, sensory afference, cognitive load

## Abstract

According to some recent empirical studies revealing that creativity is linked to sensorimotor components, the current research was aimed at evaluating whether sensory afferences could modulate originality in drawing of children and adolescents. Sixty-nine children from 1st, 3rd, 6th, and 8th grades were required to produce a man who exists and a man who doesn’t exist with fingers or stylus on a tablet and with a pen on paper. Drawings were assessed with an originality scale comparing original drawings to unoriginal ones. Since, in comparison to drawings made on paper with a pen, drawing with fingers enhances proprioceptive information, this condition was expected, according to cognitive load theory, to favor originality in drawing by reducing cognitive resources devoted to motor control of the graphic gesture (lowering intrinsic load). On the contrary, since the use of a stylus involves a proprioceptive loss of information, which enhances intrinsic load by increasing cognitive resources devoted to motor control, it was expected that drawing with a stylus on the tablet would lead to the least original drawings. Results only partially confirmed these hypotheses. While the use of fingers on the tablet led to the highest original scores, using a stylus on the tablet did not impair originality in drawing of children and adolescents. On the opposite, the use of a stylus led 3rd–8th graders to perform better than with pen on paper. This modulation of the tool on originality does not confirm the hypotheses formulated in accordance with the cognitive load framework. However, it could be explained according to an embodied perspective of creativity considering the creative process as relying on a sensorimotor prediction process in which sensory afferences are central to generating and evaluate creative ideas. This research opens new avenues on creativity and proposes to consider the development of predictive motor control as a significant part of creativity development.

## Introduction

How can new technologies enhance our knowledge of the creative process? Multiple attempts have been made to understand and describe human creativity through the years, from the mystic idea of creativity breathed by gods with Plato to the philosophical conception of the creative genuine (Duff, 1767; Galton, 1879, 1883; James, 1880). But it is only in the 1950s that creativity was investigated as a field in psychological studies (Guilford, 1956, 1967), leading around 40 years later to a consensual definition of creativity, conceived as “the capacity to realize something new and adapted to the context” (MacKinnon, 1962; Barron, 1988; Sternberg, 1988; Ochse, 1990; Sternberg and Lubart, 1991, 1995, 1996; Lubart et al., 2003). According to this definition, the creative product must be adapted to the context’s criterion and be new enough to stand out from common products. Creativity lies upon three indicators (Mouchiroud and Lubart, 2001) which are the most used for evaluating creativity: fluency (number of appropriate responses), flexibility (variability of themes in responses), and originality (novelty of responses). The latter has long been considered the main component of creativity, corresponding to the evaluation and selection of own’s idea, based on prior knowledge (Mouchiroud and Lubart, 2001; Lubart and Georgsdottir, 2004). Lubart et al. (2003) consider creativity as resulting from an interactive combination of cognitive (including convergent and divergent thinking, intelligence, knowledge), conative (personality traits and motivation), emotional and environmental (family, school) factors including new technologies, leading to different levels of creativity. This multivariate approach to creativity is to date one of the most documented and developed in experimental research.

In addition to these factors, it has recently been proposed to focus on a forgotten part of creativity: sensorimotor components. As a matter of fact, some recent studies focused on the links shared by these two processes (e.g., Nikolaeva et al., 2018; Gaggioli et al., 2019; Fleury et al., 2020). Recently, neuroscience studies highlighted the involvement of motor regions, such as the premotor cortex, the supplementary and pre-supplementary motor areas, and the posterior inferior frontal gyrus, in musical creativity (Bashwiner, 2018; Bashwiner and Bacon, 2019). The present exploratory study aims to extend this embodied perspective of creativity by focusing on sensorimotor afferences during creative drawings.

To what extent sensory afferences can modulate creative drawing executed on a digital screen? Although this question has not been investigated to our knowledge in the case of creativity, the multimedia learning theory (Mayer, 2005) usually applied to the context of digital learning could help in bringing some answers. Multimedia learning refers to any situation in which information processing occurs through two different channels, for instance, audio and visual, which is common in the digital context. Multimedia learning can be explained according to cognitive load theory (Mayer and Moreno, 2003; Sweller et al., 2011). Cognitive load theory proposes that there is a delimited amount of cognitive resources available for a specific task. In a learning task, cognitive resources can be allocated to the *essential processing* for the comprehension of the task; to the *intrinsic processing* devoted to the realization of the task, such as controlling gestures in a physical interaction learning task; or to the *extrinsic processing* to manage information primed by the design of the task but which are not essential in its comprehension, such as adding background music in a narrative learning (Mayer and Moreno, 2003; Sweller et al., 2011). Applied to a sensorimotor task in the multimedia learning context, increasing the processing demands inherent to the sensory modalities used in the task may exceed the processing capacity of the cognitive system, leading to a cognitive overload (Brown et al., 2009). As stated in Bara and Tricot (2017), this overload could be due to an increased intrinsic load, according to which the kind of movement performed during a task would make the task harder to accomplish. For example, while comparing tactile exploration of concave *versus* raised letters, Bara and Gentaz (2011) found that the strategy used by children in concave letter exploration led to higher cognitive costs than the one used in raised letter condition, resulting in less memorized concave letters than raised ones.

The rationale of the present study is the following: in order to investigate if, as suggested by recent findings, sensorimotor components are involved in creativity, then varying sensory afferences during a drawing creative task that reduce or increase the intrinsic cognitive load devoted to the motor control of the task would have consequences on originality performances. Tablets are electronic devices presenting a visual screen controlled by gestures and tactile actions executed with fingers or stylus (Dubé and McEwen, 2015). This tactile feature allows interacting very easily with the device, even at a young age, using a stylus or directly with finger (Cooper, 2005; Geist, 2012; McManis and Gunnewig, 2012; Dubé and McEwen, 2015; Piotrowski and Meester, 2018; Sakr, 2018). However, acting with fingers or with stylus on a tablet modifies afferent and efferent kinesthetic and proprioceptive information. Acting with fingers could reduce the cognitive load devoted to the motor control during the writing digital task whereas acting with a stylus would increase it. Alamargot and Morin (2015) demonstrated that compared to the roughness of a paper sheet, the smoothness of a tablet lessens the friction between the pen and the surface of the tablet which reduces proprioceptive feedback when used with a stylus. This reduction of proprioceptive feedback implies a compensating strategy to control the executing writing movement (Alamargot and Morin, 2015; Gerth et al., 2016; Guilbert et al., 2019), thus increasing the cognitive cost allocated to the motor control to complete the task – the intrinsic load. According to cognitive load theory, using a stylus during a learning task should increase the intrinsic load by requiring supplementary cognitive resources allocated to the movement control, resulting in poorer creative performances. On the contrary, acting with finger enhances the friction with the screen and therefore increases proprioceptive feedback, which should lead to a reduced intrinsic load in a learning task by facilitating motor control strategy. These variations in proprioceptive feedback consecutive to the tool used for acting on the tablet may affect differently children and adolescents as a consequence of their ability to process proprioceptive afferences. As a matter of fact, proprioception develops during childhood and adolescence as a consequence of precision improvement such that proprioception becomes more reliable over time (Holst-Wolf et al., 2016). More precisely, children younger than 8 years have some difficulty in processing proprioceptive information, leading them to rely more heavily on visual rather than proprioceptive information during an action to control that the performed movement is consistent with the desired one (Bard and Hay, 1983; Contreras-Vidal, 2006). Studies have demonstrated that variation of afferent feedback modifies writing learning abilities in preschoolers (Patchan and Puranik, 2016) as well as motor control of action (Alamargot and Morin, 2015).

Patchan and Puranik (2016) showed that using fingers on the tablet was the most efficient set thanks to an enhanced direct proprioceptive feedback on the tablet for handwriting learning. They trained 3–6 years old preschoolers to write on the tablet with fingers or with stylus, or on paper with a pen. After practicing 3 times every week for a total of 8 weeks, children wrote letters more correctly with fingers on the tablet than with stylus on the tablet or pen on paper. From these results, it could be suggested that direct contact of fingers on tablet fostered sensorimotor processing in children through the richness of proprioceptive information and favored motor learning. Results from Patchan and Puranik (2016) also showed that acting on the tablet with a stylus did not bring the same benefits as when using fingers: the sliding effect of the stylus lowered proprioceptive feedback which led to a less efficient strategy of motor control. In the same vein, Alamargot and Morin (2015) revealed that using a stylus on the smooth screen of a tablet during a writing task, compared to using a pen on paper, diminished the legibility of letters at second (6–7 years) and nine grades (14–15 years). Gerth et al. (2016) observed that in addition to preschoolers and second graders, adults were also affected by the sliding effect of the tablet’s surface when using a stylus.

Contrary to young children, older ones are able to compensate for the loss of haptic information needed for movement control when writing with a stylus on a tablet (Alamargot and Morin, 2015; Guilbert et al., 2019), by producing bigger letters (amplification of movement) and writing faster (velocity increase). This compensating strategy differs according to the age of the participants (Alamargot and Morin, 2015; Gerth et al., 2016). While 7/8 years old children increase pauses, leading to a longer duration of movements, 10/11 years old children favor increasing pen pressure and speed to maximize proprioceptive sensory input (Alamargot and Morin, 2015). Compensating strategies are tied to the developmental trend in proprioceptive processing which also induces changes in the ability of children and adolescents to use internal models to control actions.

Internal models of actions allow the brain to mimic the transformation between the motor command and sensory signals (Kawato, 1999). In order to control ongoing actions through accurate and fluent movements, we make use of feedforward processes (Miall and Wolpert, 1996) allowing a prediction of the movements to be executed as well as a prediction of their related sensory consequences. In this case, two types of internal models are used. First, inverse models are used as a controller to select an adapted motor program. Second, direct models are used as a predictor of sensory consequences of the ongoing movement, informed by a copy of the motor program issued from inverse models – the efference copy (Wolpert and Kawato, 1998). Thus, feedforward internal models are predictive representations transforming action commands into their sensory consequences. This prediction process supported by internal models of action can also be used to control off-line actions, in a simulation process, like imagined action such as motor imagery (Jeannerod, 1994) or creativity. In the case of motor imagery in which covert actions are decoupled from any actual sensorimotor interaction (Jeannerod, 1994), internal models allow simulating future or potential motor actions without realizing it (Guilbert et al., 2018).

A significant increase in the use of predictive motor control is commonly observed around 8 years (Hay, 1978, 1979; Bard and Hay, 1983; Molina et al., 2008) followed by a second one around 11–12 years (Caeyenberghs et al., 2009; Smits-Engelsman and Wilson, 2013). This means that the loss of proprioceptive feedback could be compensated by maximizing proprioceptive afferences through the use of predictive motor control from the age of 8. Consequently, Guilbert et al. (2019) observed that reducing sensory feedback affected handwriting performances more in young than older children. Older children exaggerated letter size and pen pressure to maximize the amount of proprioceptive information and made shorter pauses suggesting an increased use of feedforward motor control. After 8 years, the sliding effect of the stylus on a tablet could thus be compensated by the use of feedforward motor control of action (Kandel and Perret, 2015; Guilbert et al., 2019). These results suggest that children older than 8 May exaggerate their movement to maximize the use of proprioceptive information of the ongoing action to compensate for the loss in haptic feedback. Using a stylus reduces haptic feedback, requiring feedforward sensory prediction to compensate for the unavailable information. However, it is only after 8 years that children would be able to compensate for the loss of tactile afferences as a consequence of an improvement in predictive processes.

These results obtained for the writing task offer some interesting issues concerning creative drawings. Creativity, as a cognitive process, could be modulated by the cognitive load processes involved in learning tasks. The ability to produce original drawings could depend on the cognitive load induced by the modification of sensory afferences primed by the task. The different proprioceptive feedback induced by the use of tablets offers a particularly well-adapted situation to vary this cognitive load.

To our knowledge, only one study dealt with the question of a possible link between direct fingertip feedback on tablets and originality (Bitu et al., 2019). They invited children aged 6–7 and 8–9 to draw, based on Karmiloff-Smith (1990), “a man that exists” (unoriginal drawing) and “a man that doesn’t exist” (original drawing) with fingers on the tablet and with a pen on paper. Originality in drawings was assessed by means of a graphical scale developed by the authors, inspired by the study of Karmiloff-Smith (1990), which allowed to compute an originality score by comparing original drawings to unoriginal drawings of each child. Results showed that both 6–7- and 8–9-years old children were more original with fingers on the tablet than with pens on paper. From this study, Bitu et al. (2019) concluded a facilitator effect of finger use on a tablet for original drawing in comparison to a pen on paper. Yet, by comparing fingers on a touchscreen versus pen on paper, no conclusions could be stated about the nature of the observed benefits, which could be due to the tool (finger versus pen) as well as to the surface (touchscreen versus paper) on which drawings were produced. The use of a stylus on touchscreen was thus essential to disentangle the link between sensorimotor components and creativity. Indeed, as stated previously, while using fingers on the tablet is known to enhance tactile feedback on fingertips (Patchan and Puranik, 2016), using a stylus on a tablet leads to poorer proprioceptive feedback (Alamargot and Morin, 2015). The present study was aimed at extending this previous work by evaluating whether varying sensory afferences will impact the originality of children and adolescents drawing with finger and stylus on a tablet, and with pen on paper. To assess originality, we adapted our experiment from Karmiloff-Smith (1990) and Bitu et al. (2019) in which children operated several types of change between the drawing of a man who exists and a man who doesn’t exist. We compared originality performance of four different age groups related to the development of the capacity to generate and use internal models for the control of on-line and off-line action, at 6–7 (1st graders), 8–9 (3rd graders), 11–12 (6th graders), and 13–14 years old (8th graders). These ages correspond to periods before (1st grade), during (3rd and 6th grade), and after (8th grade) the transition commonly reported in the literature concerning the use of internal models of action.

This study addresses the effect of modifying proprioceptive feedback to modulate originality in drawings. In the light of empirical studies, two hypotheses can be drawn. If creativity is related to sensory afferences, then it was expected that drawing with fingers on the tablet would increase originality at all ages, compared to drawings made with pen on paper. Increasing proprioceptive feedback on fingertips may enhance motor control strategy and, as a consequence, would reduce the cognitive load allocated to motor control in favor of the drawing task. Second, it was expected that, compared to drawings made with pen on paper, using a stylus would decrease originality in drawings at all ages as a consequence of an increased cognitive load this situation induces to compensate for the reduced sensory feedback available to the motor control execution of the drawing task.

## Materials and Methods

### Participants

An initial sample of 70 participants was recruited in two schools located in Normandy, France. Participants were enrolled on a voluntary basis, following a convention that defined data collection, with the consent of Normandy education academy and schools, along with written authorization from their parents or legal tutor. Inclusion criteria were not met for 1 adolescent. None expressed their wish to abort the experiment before the end of the procedure, leading to a total of 69 children and adolescents aged from 6 to 14 years old (mean age = 10 years, 0 month) retained for this study. Children and adolescents in school were recruited in 1st grade (*n* = 15; mean age = 6 years, 11 months; min = 6 years, 6 months; max = 7 years, 4 months), 3rd grade (*n* = 22; mean age = 8 years, 10 months; min = 8 years, 2 month; max = 9 years, 3 months), 6th grade (*n* = 18; mean age = 11 years, 4 months; min = 10 years, 5 months; max = 12 years, 0 month) and 8th grade (*n* = 14; mean age = 13 years, 5 months; min = 12 years, 9 months; max = 14 years, 1 month).

### Materials

Digital drawings were made on a Microsoft Surface Pro 4 tablet, 12.3″ screen and 2736 × 1824 px resolution, with a Microsoft Surface Pen measuring 144 mm × 9.5 mm × 10.2 mm. Drawings were made on an app designed in our laboratory, presenting a white surface on which it can be drawn with stylus or fingers by tracing only black traits. Erasing function was not enabled on the app. Paper sheets measuring the same size as the screen (260 mm × 175 mm), and a black pencil were used. Eraser was not allowed when drawing on paper.

### Inclusion and Exclusion Criteria

For each participant, exclusion criteria were related to the task feasibility such as identified severe visual impairment (e.g., low vision, blindness) or severe motor impairment (e.g., excessive weakness), that would make the participant unable to perform the task correctly. To this end, a NEPSY-II visuomotor precision task (Korkman et al., 2014) was performed to measure graphomotor speed and accuracy during a graphical task. In this task, participants were asked to draw lines inside of tracks as quickly and accurately as possible. The number of errors and completion time are recorded in this test to compute a visuomotor precision score. The lower the score’s value, the higher the visuomotor precision level. This test allowed to control for visuomotor precision impairment that could affect a drawing task, by excluding each participant under 2*SD* from the standardized mean of its age group.

### Control Tasks

Prior to the experimentation, and after completing the NEPSY-II visuomotor task, each participant was asked to perform a functioning control task. The functioning task allowed control of children and adolescents’ knowledge of the core movements to be applied on a tablet. Participants were asked to perform eight subtasks with Instagram used offline, referring to eight core movements from the touch gesture reference guide of Villamor et al. (2010). This task’s procedure was sum up in Table 1. If a child or an adolescent was in difficulty with one of the instructions, the experimenter could help him/her by verbalizing the movement required to accomplish the given instruction (for example, “to open the app, you have to double tap”).

**TABLE 1:** Description of the functioning task procedure according to the touch gesture reference guide (Villamor et al., 2010).

Instruction	Targeted movement from Villamor et al. (2010)
Move Instagram icon on desktop	Press and drag
Start Instagram app	Double tap
Take a picture and add a smiley	Single tap
Enlarge the smiley	Spread
Shrink it	Pinch
Move it	Tap and drag
Rotate it	Rotate
Turn smiley page to see recent smileys	Flick

### Experimental Procedure

Children and adolescents were observed individually in a quiet room of the school and high school. They sat down in front of a table (700 mm × 500 mm × 750 mm) where the touchscreen or the sheet of paper was placed. Each participant had to produce three original and three unoriginal drawings of a man with fingers on the tablet, with stylus on the tablet and with a pen on paper following specific instructions. Following the procedure used by Karmiloff-Smith (1990) with pen and paper to bring creativity to drawings, children were asked to draw “a man who exists” for unoriginal drawings and “a man who doesn’t exist” for original drawings. Tool order (pen on paper, finger on tablet, and stylus on tablet) and instruction order (original and unoriginal) were systematically counterbalanced across each participant. Once a first drawing was produced, it was removed before starting the execution of a new one. During the whole experimental session, the experimenter stood near the participant to help him/her the understand the task if he/she needed to.

### Coding

A total of 414 individual electronic (276) and paper (138) drawings were collected for analysis: one half being original drawings and the other half unoriginal drawings. Unoriginal drawings were used as a baseline to assess originality considering interindividual differences in the representation of a man in a drawing. The logic was to consider as being original in the drawings, any modification operated in comparison to the unoriginal man drawn with the same tool. Originality in drawing was thus rated by comparing an original drawing to an unoriginal one made with the same tool by the same participant, allowing to score any modification operated from one condition to another with the same tool.

Several types of graphical changes were considered for the coding. As reported by Karmiloff-Smith (1990), seven categories of changes can be observed for the drawing of something (house, man, or animal) that does not exist in comparison to the drawing of something that exists as follows:

- Addition and deletion of elements (e.g., 2 heads instead of 1, or deletion of the arms), coded in our scale as the addition or deletion of elements that were already drawn on the drawing of a man who exists;
- Modification operated on the shape of elements (e.g., a triangular head instead of a circle one);
- Modification operated on the size of elements (e.g., a head two times bigger than the trunk);
- Insertion of new elements (e.g., wings added to the trunk), referring to the insertion of something that was not drawn on the drawing of a man who exist;
- Position, orientation, and perspective modifications of elements or the whole drawing (e.g., eyes and mouth inverted);
- Cross-conceptual categories modifications of the whole drawing (e.g., an animal-shaped man);
- Modifications operated on the shape of the whole drawing (e.g., a circle-shaped man).

These seven categories considered global modifications operated on the whole drawing and local modifications operated on elements of the drawing. Local modifications concerned 15 elements defined as parts constituting a man, whose presence is rated in Goodenough (1926) scale of a man drawing. Goodenough (1926) considered the head, eyes, nose, mouth, nostrils, ears, hairs, neck, trunk, arms, hands, fingers, legs, and feet. To this list, we added teeth as an element of the drawing since children could use this part of the face in an original way.

For each modification operated on elements (local modifications), 0.5 point was assigned. Each global modification was awarded the double, i.e., 1 point, since it concerned larger modifications than those consecutive to a local element modification. Only one exception to this was for “position, orientation, and perspective” category which could be rated up to 1.5 points since it could concern simultaneously local and global modifications. As an example, if a participant exchanged the mouth with an eye, it would concern at least two different elements of the “position change” category, which would result in a strongly increased originality score. This category was thus awarded 0.5 point for a local modification, no matter the number of concerned elements. One mouth and one eye exchanged would thus be awarded the same point (0.5) as if it was exchanged with two eyes. Concerning a modification of the whole drawing in position change (for example, a reversed man), 1 point could be awarded for a global modification.

To sum up, with the rating scale, the originality score calculated for each original drawing was defined by the sum of points awarded to local and global modifications made on the original drawing in comparison to the unoriginal drawing made by the same participant with the same tool. Three originality scores were thus calculated using the rating scale (Table 2), one for each original drawing executed on the touchscreen with fingers, with stylus, and on paper with a pen. The calculation of originality score in drawings was thus consistent with the definition of originality, that is, the capacity to assess and select a novel idea among all other own ideas, depending on the acquired knowledge, i.e., what children and adolescents know about the drawing of a man (Runco, 1991; Lubart and Georgsdottir, 2004).

**TABLE 2:** Rating scale used to assess originality in drawings.

Graphical change	Definition	Type of change	Awarded points (min–max)
Deletion/addition (Figure 1)	Element in the drawing of a man who exist (head, eyes, nose, mouth, nostrils, teeth, ears, hairs, neck, trunk, arms, hands, fingers, legs, feet) replicated or deleted	Core element	0–7.5
Shape of elements (Figure 2)	Element (head, eyes, nose, mouth, nostrils, teeth, ears, hairs, neck, trunk, arms, hands, fingers, legs, feet) whose shape was different from the drawing of the man who exist	Core element	0–7.5
Size of elements (Figure 3)	Element (head, eyes, nose, mouth, nostrils, teeth, ears hairs, neck, trunk, arms, hands, fingers, legs, feet) whose size was different from the drawing of a man who exist	Core element	0–7.5
Insertion of new elements (Figure 4)	New element (head, eyes, nose, mouth, nostrils, teeth, ears hairs, neck, trunk, arms, hands, fingers, legs, feet) that were not present on the drawing of a man who exist	Core element	0–7.5
Position, orientation, and perspective (Figure 3)	Elements (head, eyes, nose, mouth, nostrils, teeth, ears, hairs, neck, trunk, arms, hands, fingers, legs, feet) and/or the whole drawing different in position, orientation, and perspective from the drawing of a man who exist	Core element and on the whole	0–1.5
Cross-conceptual category (Figure 5)	Whole drawing presenting insertions crossed with other conceptual categories (trees, animals, technology, …)	On the whole	0–1
Form of the whole (Figure 5)	Drawing whose whole form was differently shaped in comparison of the drawing of a man who exist	On the whole	0–1
		TOTAL	0–33.5

The originality score could be rated up to 33.5 points for each original drawing but since the originality scale aims to cover a large range of modification possibilities in order to capture each modification operated on the original drawing, a single original drawing will most probably use only a few of this range of possibilities, leading to relatively low scores on the 33.5 total available points.

### Data Analysis

To ensure that instructions were well understood, an interrater agreement score was calculated with a random sample of 100 unoriginal and original drawings produced in the three conditions (finger on tablet, stylus on tablet, and pen on paper). Two naive observers were asked to categorize the drawings as being original or unoriginal. Cohen’s Kappa showed a strong inter-reliability agreement between the two raters (*K* = 0.91), showing that even if originality can be quite subjective, the instruction given to adolescents led them to produce original drawings.

Prior to any statistical analysis, a Mauchly’s sphericity test (*p* = 0.237) allowed to perform a repeated measures ANOVA, with originality score as a dependent variable, tool (pen on paper, stylus on tablet, finger on tablet) as repeated factors, and grade (1st, 3rd, 6th, or 8th grade) as between-subject factor. In addition, we performed Cochran analyses to investigate whether the occurrence of graphical transformations varied according to the drawing conditions.

**FIGURE 1:**
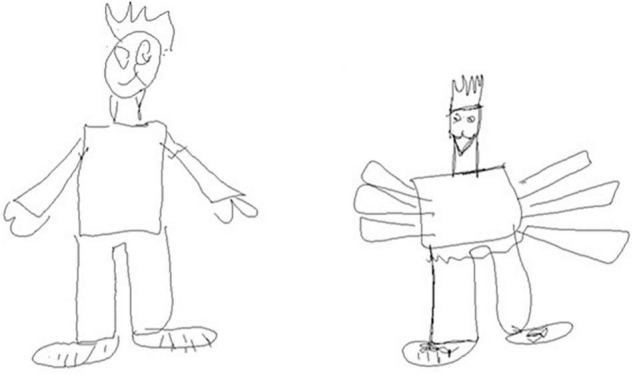
Example of addition and deletion of elements in stylus drawing on tablet at 6th grade: compared to the unoriginal drawing (on the left), arms have been replicated and hands have been deleted in the original drawing (on the right).

## Results

A first ANOVA analysis was conducted in order to control for the potential exhausting effect of the procedure, by testing the effect of the instruction order (original then unoriginal; unoriginal then original) and of the tool order (pen-finger-stylus; pen-stylus-finger; stylus-pen-finger; stylus-finger-pen; finger-stylus-pen; finger-pen-stylus) on originality scores. Results showed no significant effect of instruction order on the originality scores, *F*(2,114) = 0.854, *p* = 0.42 η_p_^2^ = 0.15, neither of tool order on originality scores *F*(10,114) = 0.223, *p* = 0.98. These results indicate that systematic counterbalancing was enough to thwart the exhausting factor of producing six drawings in raw. Therefore, instruction order and tool order were then excluded from the main analysis.

**FIGURE 2:**
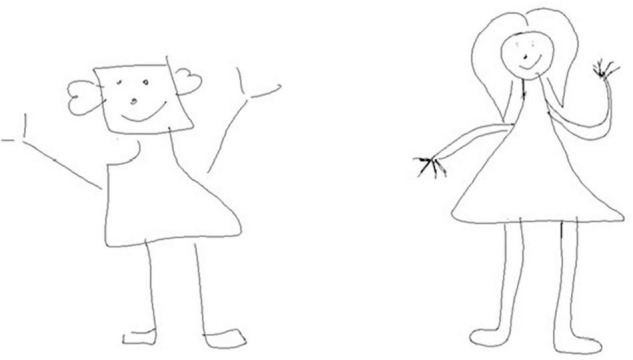
Example of shape modification of elements in stylus drawing on tablet at 6th grade: compared to the unoriginal drawing (on the left), the shape of the head and of the trunk has been modified in the original drawing (on the right).

**FIGURE 3:**
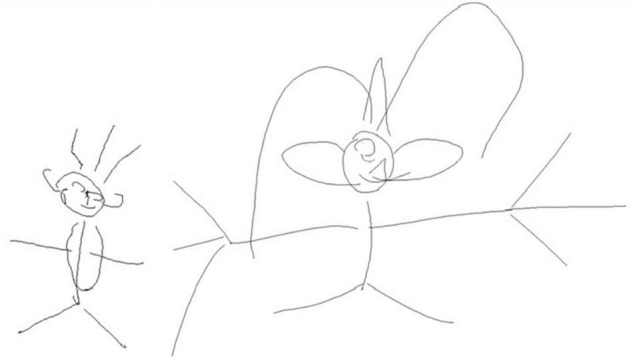
Example of size modifications on elements and orientation modification in finger drawing on tablet at 3rd grade: compared to the unoriginal drawing (on the left), the size of the ears and hairs have been modified, and eyes orientation changed in the original drawing (on the right).

**FIGURE 4:**
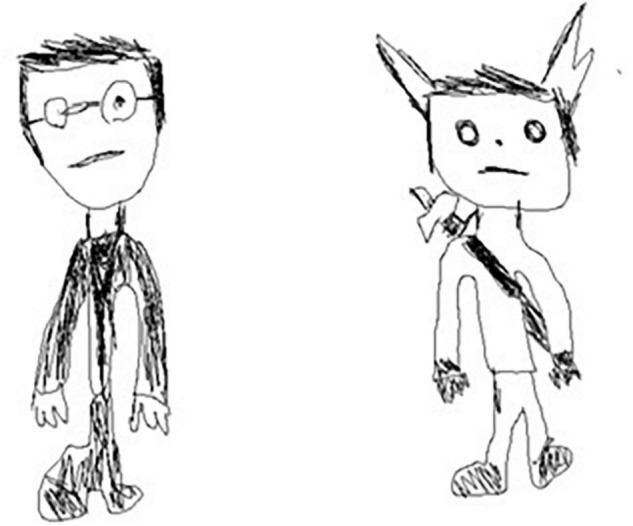
Example of insertion of novel elements in stylus drawing on tablet at 6th grade: compared to the unoriginal drawing (on the left), ears have been added to the original drawing (on the right).

We then performed an ANOVA to analyze the effect of varying proprioceptive information with different tools (finger on tablet, stylus on tablet, pen on paper) on originality scores in the 4 groups of children (1st, 3rd, 6th, and 8th grade). Results showed a significant effect of the grade, *F*(3,65) = 3.83, *p* = 0.01 η_p_^2^ = 0.15, such that, as shown on Figure 6, originality scores increased from the 1st (*M* = 2.59, SD = 1.41), 3rd (*M* = 3.23, SD = 1.55), 6th (*M* = 3.65, SD = 1.30) to the 8th grade (*M* = 3.87, SD = 1.71). *Post hoc* Bonferroni comparisons validated this increasing trend, 8th graders scoring significantly higher than 1st graders (*p* = 0.018).

**FIGURE 5:**
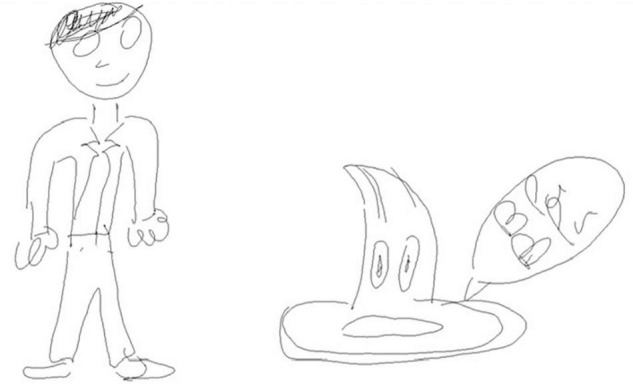
Example of modifications operated on the form of the whole and of cross-conceptual categories insertion in finger drawing on tablet at 6th grade: compared to the unoriginal drawing (on the left), the whole original drawing (on the right) was differently shaped and included cross-category insertion such that the man was represented as a hat.

**FIGURE 6:**
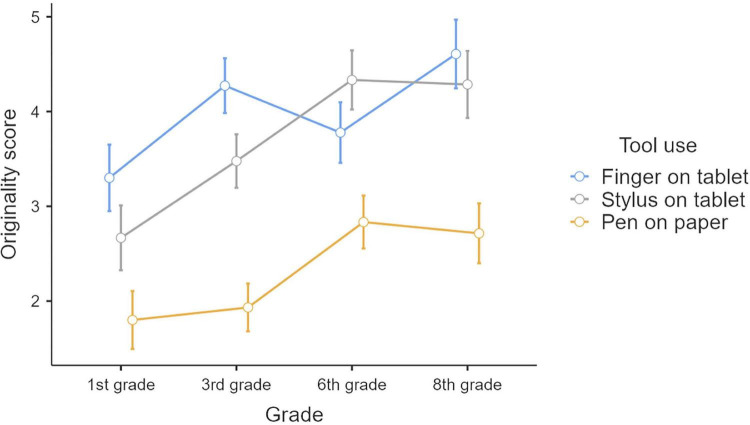
Originality scores (mean and standard errors of mean) according to the tool used and grade.

A significant effect of the tool used on originality scores was also observed, *F*(2,130) = 85.43, *p* < 0.001, η_p_^2^ = 0.57. As shown in Figure 6, originality scores were higher for drawings produced on tablet with finger (*M* = 4.00, SD = 1.41) and on tablet with stylus (*M* = 3.69, SD = 1.45), compared to drawings produced with pen on paper (*M* = 2.30; SD = 1.24). *Post hoc* Bonferroni comparisons confirmed that finger and stylus were not significant (*p* = 0.148), while significant differences were found between finger and pen (*p* < 0.001) and stylus and pen (*p* < 0.001).

Finally, the ANOVA also revealed an interaction between the tool used and the grade factor, *F*(6,130) = 4.11, *p* < 0.001, η_p_^2^ = 0.16, such that, although originality scores increased with the school grade and were higher for drawings produced with finger than with pen, an exception was observed for the 1st grade. As shown in Figure 6 and Table 3, while 3rd, 6th, and 8th graders performed better with fingers (*p* < 0.001) and with stylus (*p* < 0.001) compared to pen on paper, 1st graders performed better only with fingers compared to pen on paper (*p* < 0.001), stylus compared to the pen being non-significant (*p* = 0.111).

**TABLE 3:** Descriptive analysis of originality scores according to the grade and tool used.

		Finger on tablet	Stylus on tablet	Pen on paper
1st grade	N	15	15	15
	Mean	3.30	2.67	1.80
	Median	3.00	3.00	1.50
	SD	1.37	1.32	1.18
	Range	4.50	4.50	4.50
	Min–Max	1.50–6	0.50–5.00	0.50–5.00
3rd grade	N	22	22	22
	Mean	4.27	3.48	1.93
	Median	4.25	3.50	2.00
	SD	1.34	1.29	1.03
	Range	5.50	5.50	4.50
	Min–Max	2.00–7.50	1.00–6.50	0.00–4.50
6th grade	N	18	18	18
	Mean	3.78	4.33	2.83
	Median	3.75	4.50	2.50
	SD	1.17	1.10	1.21
	Range	4.50	3.50	4.50
	Min–Max	2.00–6.50	2.50–6.00	1.00–5.50
8th grade	N	14	14	14
	Mean	4.61	4.29	2.71
	Median	4.50	4.25	2.50
	SD	1.57	1.63	1.37
	Range	6.00	6.00	4.50
	Min–Max	1.50–7.50	1.00–7.00	0.50–5.00

In order to analyze the degree to which each item was used to modify the drawn man in each condition (finger, stylus, and pen), a second analysis was conducted using Cochran’s Q test. This analysis was aimed at determining whether a drawing condition favored more specifically a category of transformation. First, Table 4 shows that all elements of the man being confounded, each category of transformation was used in each condition, meaning that each tool is likely to elicit each type of modification. Results also revealed that each type of modification was not equally used. Participants made more addition or deletion [*Q*(2) = 35.18; *p* < 0.001], insertion [*Q*(2) = 9.91; *p* = 0.007], and modified more frequently the shape [*Q*(2) = 40.71; *p* < 0.001] and size [*Q*(2) = 17.31; *p* < 0.001] of elements when drawing with finger or with stylus on tablet, than with a pen on paper. Thus, although each category of transformation was observed whatever the support used, transformations were more used when drawing on a tablet with fingers and stylus than on paper with a pen, all elements confounded.

**TABLE 4:** Occurrence (percentage) of each item of originality scale as a function of drawing condition (significant differences on Cochran’s Q test are in bold).

	Finger	Stylus	Pen
I – Deletion or addition of elements			
**0 - All elements confounded**	**15.49**	**13.86**	**8.6**
1- Head	5.8	4.35	0
2- Eyes	15.94	14.49	13.04
**3- Nose**	**21.74**	**28.98**	**11.59**
4- Mouth	20.29	10.14	11.59
5- Nostrils	0	1.45	0
6- Teeth	0	4.35	2.9
**7- Ears**	**4.35**	**11.6**	**2.9**
8- Hairs	26.09	27.54	23.2
**9- Neck**	**24.64**	**15.94**	**4.34**
10- Tronk	5.8	4.35	0
11- Shoulders	0	0	0
12- Arms	18.85	18.85	10.14
13- Hands	18.84	15.94	11.59
**14- Fingers**	**37.68**	**33.33**	**11.59**
15- Legs	23.19	13.04	15.94
16- Feet	25.64	17.4	18.84

II – Shape of elements			

**0 – All elements confounded**	**22.28**	**21.37**	**14.49**
**1- Head**	**3.68**	**26.09**	**15.94**
**2- Eyes**	**59.42**	**50.72**	**31.88**
3- Nose	8.69	14.49	5.8
4- Mouth	30.43	40.58	33.33
5- Nostrils	0	0	0
6- Teeth	0	1.45	1.45
7- Ears	0	2.9	1.45
8- Hairs	43.48	43.48	34.8
**9- Neck**	**15.94**	**10.14**	**5.8**
**10- Trunk**	**53.62**	**47.83**	**33.33**
11- Shoulders	0	0	0
**12- Arms**	**34.8**	**36.23**	**11.6**
13- Hands	4.35	5.8	1.45
14- Fingers	10.14	10.14	11.6
15- Legs	39.13	33.33	23.18
16- Feet	18.85	18.85	20.29

III – Size of elements			

**0 – All elements confounded**	**4.35**	**3.89**	**1.53**
1- Head	13.04	13.04	5.8
2- Eyes	1.45	2.9	0
3- Nose	4.35	1.45	0
4- Mouth	1.45	1.45	0
5- Nostrils	0	0	0
6- Teeth	0	0	0
7- Ears	1.45	1.45	1.45
8- Hairs	1.45	0	0
9- Neck	4.35	8.7	2.9
10- Tronk	11.59	4.35	4.35
11- Shoulders	0	0	0
12- Arms	13.04	10.14	4.35
13- Hands	1.45	1.45	1.45
14- Fingers	2.9	1.45	1.45
15- Legs	11.6	11.6	2.9
16- Feet	1.45	4.35	0

IV – Insertion of new elements			

**0 – All elements confounded**	**5.34**	**5.8**	**3.26**
1- Head	7.25	10.14	5.8
2- Eyes	1.45	1.45	1.45
**3- Nose**	**11.59**	**4.35**	**1.45**
4- Mouth	1.45	7.25	4.35
5- Nostrils	1.45	1.45	0
6- Teeth	5.8	1.45	5.8
**7- Ears**	**13.04**	**10.14**	**2.9**
8- Hairs	14.5	5.8	8.7
9- Neck	4.35	7.25	0
**10- Tronk**	**0**	**8.7**	**4.35**
11- Shoulders	0	0	0
12- Arms	1.45	2.9	5.8
13- Hands	5.8	8.7	2.9
14- Fingers	5.8	8.7	4.35
15- Legs	7.25	7.25	1.45
16- Feet	4.35	7.25	2.9

V – Position, orientation, and perspective modifications			

1- On the whole	0	0	0
2- On elements	2,9	0	1,45

VI – Cross-conceptual categories modification			

On the whole	4,35	4,35	2,9

VII – Form of the whole			

**On the whole**	**18,84**	**2,9**	**2,9**

Then, we performed Cochran’s Q test on each category of transformation for each element considered for the drawing of a man (see Table 4) according to drawing conditions (finger on tablet, stylus on tablet, and pen on paper). Among the 15 elements constituting the man, shoulders were never modified, and nostrils were very few modified (< 5% in each category). All other elements were modified in the three drawing conditions. However, Cochran’s Q test revealed some differences in the degree to which each element was modified according to the tool used. Results showed that nose addition or deletion [*Q*(2) = 7.52; *p* = 0.02], neck addition or deletion [*Q*(2) = 12.87; *p* = 0.002], and finger addition or deletion [*Q*(2) = 16.41; *p* < 0.001] were more used with finger and stylus on tablet than with pen on paper, as well as eyes shape [*Q*(2) = 12.87; *p* = 0.002], trunk shape [*Q*(2) = 8.66; *p* = 0.01], and arms shape [*Q*(2) = 16.06; *p* < 0.001]. In addition, head shape [*Q*(2) = 9.66; *p* = 0.008], neck shape [*Q*(2) = 6.72; *p* = 0.03], and nose insertion [*Q*(2) = 7.09; *p* = 0.03] were more used only by finger on tablet than with pen on paper, and ears addition or deletion was more used with stylus on tablet than with finger on tablet or pen on paper, *Q*(2) = 6.20; *p* = 0.04. The form of the whole was more modified with finger on tablet than with stylus on tablet or pen on paper, *Q*(2) = 16.13; *p* < 0.001.

## Discussion

The present study was aimed at determining if the modification of sensory afferences could affect originality in drawings produced by children and adolescents aged 6–14.

First, control analyses proceeded on the originality scale revealing that all categories of modification were used by participants with each tool, all elements confounded. This result means that the scale allowed for assessment modifications operated with each tool used, and thus didn’t favor the scoring of originality in a drawing condition over another. Therefore, items used to assess originality were modified as well on the tablet with fingers or stylus as on paper with a pen. This analysis also revealed that modifications were higher on the tablet with fingers and stylus than with pen on paper. These higher originality scores on the tablet with finger or stylus are not tied to one specific category or element but reflect the overall occurrence of original modifications that were generally lower in pen on paper condition than with finger on the tablet or with stylus on the tablet. Thus, varying sensory afferences with the use of tablets seem to have led to globally more modifications.

If creativity is related to sensory afferences, then it was expected that drawing with fingers on the tablet would increase originality at all ages, compared to drawings made with pen on paper. Our first hypothesis stated that increasing proprioceptive feedback on fingertips could enhance motor control, resulting in a reduced cognitive load allocated to motor control in favor of the drawing task. Thus, we expected that enhancing proprioceptive feedback on fingertips when drawing on the tablet with fingers would increase originality at all ages in comparison to the use of a pen on paper.

The results from the present study confirm our first hypothesis, such that increasing haptic afferences to fingertips increased originality. As a matter of fact, children and adolescents produced more original drawings when drawing with fingers on the tablet rather than with pen on paper as revealed by higher originality scores obtained from drawing with fingers on the tablet. This result observed in adolescents up to 14 years extends the previous ones obtained in children aged 5–6 and 7–8 years demonstrating that drawing with fingers on the tablet led to more originality in drawings compared to the use of pen on paper (Bitu et al., 2019). Using fingers to draw on the tablet increased the friction between finger and screen leading in turn to an increase in the amount of available proprioceptive information. Drawing with fingers leads to an enrichment of sensory feedback to fingertips that, in turn, enhances the creative process during a drawing task from 1st to 8th grade. This effect was observed both before and after 7 years old: increasing proprioceptive afferences enhanced the originality of children aged 7 years and more, who are able to make appropriate use of proprioceptive feedback to correct their ongoing movement (Holst-Wolf et al., 2016), but also of younger ones who do not accurately use proprioceptive feedback for online control of movements (Bairstow and Laszlo, 1981; Laszlo and Bairstow, 1984). Consequently, it could be suggested that amplifying proprioceptive information may have facilitated children’s creative process by reducing the cognitive load allocated to the motor control (intrinsic load), in favor of more cognitive resources for the creative process (essential load).

Conversely, using a stylus on the tablet is known to induce a sliding effect which decreases the friction between the stylus and the screen, leading in turn to a decrease in available proprioceptive afferences (Alamargot and Morin, 2015). This decrease in available proprioceptive afference could impair the cognitive process involved in the task, by increasing the intrinsic load with resources that can’t be allocated for the essential processing, i.e., producing original drawing. Our second hypothesis stated that using a stylus on tablet would decrease originality in drawings at all ages as a consequence of the higher cognitive load induced by this situation. Thus, we expected that reducing proprioceptive feedback when drawing with a stylus on tablet would decrease originality at all ages in comparison to the use of a pen on paper. This second hypothesis was not validated. We observed that 1st graders performance when drawing with a stylus on tablet were similar to pen on paper. Lowering the available proprioceptive feedback in a drawing task did not affect 1st graders, who are known to struggle with the use of accurate proprioceptive information from the use of stylus on the tablet. It could be suggested that these young children may have used compensating strategies based on a greater mobilization of the visual component in accordance with empirical data reporting that young children are known to rely more heavily on visual rather than proprioceptive information during an action (Bard and Hay, 1983; Contreras-Vidal, 2006). In accordance with results reported by Guilbert et al. (2019) with 2nd graders, using a tablet with stylus may have enhanced visual information compared to a pen on paper, leading young children to pay more attention to the tracing that could, in turn, lead to a more intense analysis of the traced shapes and increase creativity. However, as suggested by cognitive load theory, paying more attention to the gesture should have deteriorated the cognitive resources allowed for the original task.

On the contrary, from 3rd to 8th grade, children and adolescents obtained higher originality scores when drawing with a stylus than when drawing with a pen. By 8/9 years, children were affected by the use of a stylus on a tablet, but in a positive way, such that it strengthened their originality performance. This unexpected effect could be explained as a consequence of more accurate use of afferent proprioceptive information by 8/9 years, which, along with visual information, leads to a significant improvement in predictive motor control (von Hofsten and Rösblad, 1988; Chicoine et al., 1992). As reminded in the introductive section, this improvement leads to a switch from a feedback motor control where children make pauses to control their movement with visual verification, to a feedforward motor control relying more accurately on the movement prediction. Consequently, by 8/9 years, the sensory prediction could be used to compensate for the loss of proprioceptive afferences. However, if 3rd to 8th grade children and adolescents had compensated for the initial loss of proprioceptive information with the use of feedforward motor control to make sensory predictions, this compensating strategy should have, according to the cognitive load theory (Sweller et al., 2011), increased the intrinsic load leading to a reduced cognitive resource allocated to the original task. The opposite was observed.

Altogether, the obtained results revealed that originality scores varied according to the proprioceptive afferences children and adolescents had to deal with during the drawing task. Results obtained with the stylus revealed a modulation of the tool on originality according to the age of the participants that do not fit in the hypotheses formulated with the cognitive load framework. Why did children and adolescents from 3rd grade and older draw more original drawings with stylus on tablet compared to pen on paper?

The embodied perspective of creativity could bring some arguments in the understanding of this increased performance induced by the use of a stylus in a creative drawing task. By enhancing proprioceptive information used in the prediction process, the feedforward motor control may have supported prediction possibilities involved in creativity (Dietrich and Haider, 2015). Indeed, the present results could be interpreted according to the theoretical model of creativity recently proposed by Dietrich and Haider (2015) who conceived creativity as an abstract form of sensorimotor prediction run by internal models of motor control of action (Wolpert et al., 1995; Miall and Wolpert, 1996; Kawato, 1999; Wolpert and Ghahramani, 2004). In the case of creativity, internal models would allow to generation and evaluate new and original ideas, on a trial-and-error basis, by chaining multiple internal model loops. In each of these loops, direct models would allow the generation of various ideas to reach a creative goal sent by the efference copy from inverse models. Generated ideas would be compared to the creative goal and, when they are not enough new or adapted to the context, are used as a source of information for a new loop iteration until the comparator selects the most original and adapted idea. The creative process would thus be a predictive mechanism rooted in sensorimotor activity in which sensory consequences of motor commands play a fundamental role. As a matter of fact, sensory inputs feed internal models to validate or invalidate the selected motor programs, by comparing sensory afferences available in the task to the sensory prediction. Applied to creativity, sensory afferences used in internal models would allow for validation or invalidation of each variation of ideas, by comparing sensory afferences available in the task, to the sensory prediction. Sensory afferences would thus feed multiple iterations of the internal model loops, allowing to generate of idea variations, and selecting the one the more adapted to the context. In this way, creativity would be rooted, as suggested by Dietrich and Haider (2015), in sensorimotor control of the action. Consequently, modifying sensory feedback with finger and stylus may have impacted the mobilization of the originality process: the sensory afferences specifically generated in a given action space (stylus on tablet or finger on the tablet) may have modulated originality by delimiting the range of possible outcomes, as well in generating as in selecting ideas. As reminded in the section “Introduction,” up to 7/8 years, children felt some difficulties in using accurate proprioceptive afferences. By 8/9 years, they demonstrated an improvement in processing proprioceptive information which led to an increase in the use of feedforward motor control strategy to maximize sensory input (Guilbert et al., 2019). This improvement could be the reason why children’s originality in stylus on tablet drawing was significantly higher than with pen on paper.

The present research revealed the last result that was not expected and could be in line with the previous idea. Whether drawings were made on the tablet or paper, originality scores among age groups showed an increasingly linear trend. This result partially confirms the first observation of Torrance (1968) who described creativity development as following a linear trend. However, Torrance (1968) added that 3 slump periods accompanied this positive progression, identified at 5 years, 9–10 years, and 13–14 years. He suggested that these three slump periods would be linked to changes occurring during the scholar course, such that children have to comply with the new scholar normative environment in which they are enrolled (Torrance, 1968). Contrary to Torrance, we did not observe these slumps. This could be explained according to the age of the participants (6–7 years, 8–9 years, 11–12 years, and 13–14 years) which are critical periods identified in the development of predictive motor control. As mentioned in the section “Introduction,” these age groups correspond to periods before (1st grade), during (3rd and 6th grade), and after (8th grade) the transition concerning the use of internal models of action. From the present result, we reported and in regard to the several theories discussed, it could be suggested that the development of creativity overlaps the development of predictive motor control. Future research should therefore focus on creative development through the lens of predictive motor control, for example by tracking kinematics changes in comparison to creative assessment throughout childhood.

Several limitations can be noted in our study. First, we did not consider participants’ daily use of technologies, which could have favored some children over others in the use of tablets in comparison to paper. However, Bitu et al. (2019) addressed a survey of parents with the aim of controlling this variable and found no effect of technology uses on originality gain in a drawing task on the tablet. Moreover, interacting with tablets involves only a few formal knowledge and simple gestures (Dubé and McEwen, 2015). For this reason, we used a functional task to make sure participants were able to use the basic gestures needed for the interaction on the tablet. Second, several factors pointed out by the multivariate approach to creativity (Lubart et al., 2003) could have played a role in explaining our results. More specifically, motivation may have been a confounding factor in the present study which could explain part of the difference obtained in originality scores between tablet and paper. Indeed, for children and adolescents, using tablets induce better motivation and engagement in the task (Clark and Luckin, 2013; Dubé and McEwen, 2015). More specifically, the easiness of use of tablet screens and the physical interaction consecutive to the tactile feature intensely engage children and adolescents in a multimodal task, leading to a better motivation (Amadieu and Tricot, 2014; Crescenzi et al., 2014; Geist, 2014; Dubé and McEwen, 2015). Further studies should isolate this component to determine the part of benefits due to motivation and engagement induced by the tools used.

To conclude, this study contributes to a better understanding of the process of creativity. This first empirical study highlights the crucial role played by sensory afferences in creative thinking. Moreover, it opens new avenues on the understanding of creativity that could be investigated as a predictive process rooted in predictive motor control as suggested by Dietrich and Haider (2015). Future studies should investigate this new issue.

## Data Availability Statement

The raw data supporting the conclusions of this article will be made available by the authors, without undue reservation.

## Ethics Statement

Ethical review and approval was not required for the study on human participants in accordance with the local legislation and institutional requirements. Written informed consent to participate in this study was provided by the participants’ legal guardian/next of kin.

## Author Contributions

FB: conception and design of the work, acquisition, analysis and interpretation of data for the work, and drafting and revising the manuscript. BG-M: conception and design of the work and critical advices. MM: conception and design of the work, interpretation of data for the work, and drafting and revising the manuscript. All authors contributed to the article and approved the submitted version.

## Conflict of Interest

The authors declare that the research was conducted in the absence of any commercial or financial relationships that could be construed as a potential conflict of interest.

## Publisher’s Note

All claims expressed in this article are solely those of the authors and do not necessarily represent those of their affiliated organizations, or those of the publisher, the editors and the reviewers. Any product that may be evaluated in this article, or claim that may be made by its manufacturer, is not guaranteed or endorsed by the publisher.
